# Report on Dental Progress

**Published:** 1858-03

**Authors:** J. Richardson, J. Taft, H. R. Smith


### REPORT ON DENTAL PROGRESS.

TO THE MISSISSIPPI VALLEY ASSOCIATION OF DENTAL SURGEONS.

Gentlemen:—The undersigned members of your committee on Dental Progress would respectfully report, that they have, as far as practicable, endeavored to present for your consideration such material facts and statements as relate to the subject referred to them.

In reviewing briefly the various means and instrumentalities more recently concerned in the development of Dental Science, the committee are conscious of the difficulties naturally incident to the attempt to so analyze or separate these various agencies that they may be able discriminately to present those only that clearly seem to indicate *progress.* While much that enters into the aggregate of professional attainments will be readily recognized and taken as undoubted evidence of solid advancement and practical improvement, there are, on the other hand, many elements entering into this common fund, either of questionable utility or positively worthless. That the profession is steadily advancing in usefulness, in honor and in influence is patent to all. The unsatisfied and restless spirit of inquiry, enterprise, and invention is gradually appropriating new materials in science and art, unfolding new truths and principles, and striking out new and untried paths in practice.

Prominent among the evidences of true progress in the profession, is the increasing recognition, on the part of its members, of the paramount value and importance of *asscia-tions*, and the general endorsement, either in sentiment or by actual membership, of the aims and policy of the various dental organizations scattered throughout the country, as well as their rapid increase, are substantial proofs of the fact stated.

The American Convention, so far as the history of its *past* is concerned, promises for the future a wider range of influence and usefulness than was ever enjoyed by the parent society from which it sprung. Founded upon a more liberal basis and contemplating objects and purposes more strictly in accordance with the spirit of the age and the increasing wants of the profession, it can not fail to promote, in the highest degree, a more free and extended interchange of opinion upon all topics of discussion, a greater freedom of social and fraternal intercourse, to become a more effective means of suppressing empyricism by a generous recognition and endorsement of merit or excellence, wherever found in its democracy of talent. The practical encouragement and support already extended to it by a large class of practitioners, heretofore indifferent or opposed to all organizations of the kind, furnishes a gratifying assurance that it will continue to so grow in popular favor as to become practically, and, in fact, what its name imports, a national convention. Its next meeting will be held in this city on the first Tuesday in next August, where it is earnestly hoped that its representation of members will be largely augmented.

The rapid multiplication of State and local societies constitutes another important element of progress. In something more than twelve months, no less than seven dental associations have sprung into a healthful and vigorous existence. We have the "Western Dental Society," organized in April, 1856; the "North Carolina Dental Society," in October, 1856; the "St. Louis Dental Society," in December, 1856; the "Pennsylvania Central Society of Dental Surgeons," in May, 1857; "South Jersey Dental Association," July, 1857; the "Dental Convention of Northern Ohio," in November, 1857; and the "New York Dental Society," in December, 1857.

There are now in the United States, as far as the committee are advised by published reports, ten dental societies, including, with those just mentioned, the "American Dental Convention," the Pennsylvania Association," and the "Mississippi Valley Association."

The "Mississippi Valley Association" is the oldest dental society now in existence, having been organized in 1844, the "Pennsylvania Association" following in 1845.

The committee does not propose to consume time with separate commentaries upon the condition, aims, and prospects of these several State and local societies, but will state briefly what is verified by the history of their proceedings, that they are amply encouraged by the more intelligent and appreciative practitioners of the country, that their proceedings are characterized by singular harmony, that they are effective in accomplishing the purposes contemplated in their creation, and are exerting, beyond a peradventure, a salutary and wide-spread influence in the cause of dental progress.

Indeed it would be difficult to estimate the benefits accruing from these periodical re-unions, or the extent of their influence upon the interests of the profession. Much may be hoped for in the cordial co-operation of earnest working men, recognizing a common brotherhood and cemented by a community of interests, each impelled by a generous impulse to contribute to the common fund whatever is practicable in theory or valuable in experience. These associations tend largely to the elimination of practical good. They invite comparison of individual methods of practice, and by consequence stimulate to greater excellence in practice; they encourage the free discussion of principles, and thereby develope new truths; they exalt the standard of professional deportment by placing a premium upon professional ethics; they dissipate local jealousies and personal discords, by the harmonizing influence of social contact; they cultivate increased respect for, and toleration of, opinion and engender a cordial reciprocity of good feeling and good fellowship, and should receive, as doubtless they will, the hearty support and encouragement of every friend of progress in legitimate dentistry.

The committee has nothing specially to report in relation to dental colleges. While there has not, in any case, perhaps, been any considerable increase in the attendance of students upon the several schools, we deem it sufficient cause of encouragement, that in despite of the disabling causes connected with the late disastrous and protracted financial crisis pervading all parts of the country, they have been able to maintain their ground with but little or no diminution in the number of matriculants. We do not conceive it necessary in this connexion to call your attention at any length to the importance of this means of professional advancement or the bearing of these institutions upon the interests of Dental Science. The system of collegiate instruction is too well grounded in the favor and confidence of the profession, and its claims too generally recognized to require discussion in this place.

There has been no recent increase in the number of dental periodicals.* "The "American Journal of Dental Science," the "Dental News Letter," the "Dental Obturator," and the "Dental Register of the West," comprise the list of American journals now published. These are all conducted with ability, and are well sustained by the profession. Like the arterial ramifications of the body, they are constantly pouring into all parts of the dental organism a rich and swelling tide of life-giving elements for its more perfect growth and development.

Nothing has been added to the list of elementary works or text books, since the publication of Harris' American edition of Fox.

The progress made in the operative department of dentistry is probably more difficult to delineate than that of either the *scientific*, *literary*, or *mechanical* departments. Of course, these departments can not be considered as

---

*A new quarterly, published at St. Louis, called the "American Dental Review," and edited by Dr. A. M. Leslie, has just made its appearance, and is likely, under its present management, to become a useful and popular journal.

independent of each other; they are intimately connected together, and constitute parts of a harmonious whole.

Operative dentistry is made up of minute particulars to such an extent, that to the superficial observer they seem scarcely worthy of close attention; in almost every thing, and especially here, great things are made up of small things.

In the remarks on this branch of the subject, we propose nothing more than a reference to some particulars, indicating as we think, that some advance has been made in this department.

We think it quite apparent that there is an increasing interest taken in the study of dental anatomy, physiology, and pathology. In regard to dental pathology, there has been much difference of opinion, and consequently much hypothetical reasoning. There is, doubtless, considerable progress making with the profession generally, in regard to the pathological conditions of the teeth. This knowledge is very important to the dental practitioner; without it, his best efforts are but strokes in the dark. The treatment of these conditions is vastly different from, and more efficient, than that formerly employed. At one period, almost all dentists filled teeth as though they were inorganic bodies, and no more susceptible of disease than a pebble. While it is true that such men as Koecker, Hayden, Greenwood, and Parmley did recognize the pathological conditions of the teeth, and made their operations conform to the indications thus pointed out, the great mass of operators at that time, and indeed long subsequent to it, gave no attention whatever to these things. When a tooth was found with an exposed pulp, the direction was, "*take it out.*" If a case was presented in which the dentine was so sensitive as to forbid an operation, the pulp was pronounced exposed and the tooth extracted or permitted to remain, with the hope that it would decay away as soon as possible. The days of such ignorance, with the mass of the profession, have passed away.

The first thing that is noticed now with the majority of our operators, is the conditions that exist in any given case, modifying the treatment and operations to the existing indications, seeking to change or modify unfavorable conditions by local or general treatment. All this is preparatory to the operation itself, but it is, in many cases, equally important.

Your committee are pleased to entertain a reasonable hope that the profession will go on to higher and higher attainments, until, in this pacticular, there shall be nothing more to do except to properly train those about to enter the ranks of the profession.

The subject of filling teeth is one of vast interest, not only to the dental profession, but to all who have, or expect to have decayed teeth; and any thing that will tend, in any respect, to improve this operation should be noted and employed by the profession.

One point in the operation of filling, to which attention is more closely directed than formerly, is the formation of cavities. Until recently there seemed to be no definite idea in regard to the form of individual cavities. Formerly, upon inquiry of operators generally, as to the forms of individual cavities, the reply was, "Oh, I remove all the decay and have the cavity of such a form as to retain the gold, larger within than at the orifice," a very good form of course, but too general to be of value to any one, and especially to a young practitioner. Now, when inquiry is made of a good operator, one who is posted up in his profession, in regard to his manner of forming cavities, he forthwith specifies a cavity, its location and extent, and than describes minutely, the exact form he would give each wall of the cavity, the particular angles desirable and their location; the best location for retaining points and the best manner of forming them. Thus all parts of the operation is so described that it is perfectly comprehensible.

The descriptions we got a few years ago of dental operations were somewhat like the man's description of a steam engine: "a boiler, a cylinder, a piston rod, a fire-place, and a fly wheel, so arranged as to give rotary motion to the fly-wheel." Not a very satisfactory description to one who wished to build an engine.

In regard to the forms and conditions of gold for filling, we think there has been much improvement. There are now principles employed and made available, that but a little while ago were wholly overlooked. The cohesive property of gold was not made available, as a general thing, until within the last two or three years. That is considered by all who have become familiar with its use as a very great and important step in dental practice.

There are two forms of gold in which this principle is made available, viz: annealed or adhesive foil, and crystal gold. This principle was for a long time rejected, owing to a want of knowledge and skill in its management. Formerly it was necessary to have a cavity larger within than at the orifice, and admissible only to bring the filling flush with the borders of the cavity, and then, unless it was skillfully deposited, it would escape in fragments. Now, with adhesive gold, a plug can be made almost any where in any kind of a cavity,—there is no such thing as a cavity in a decayed tooth, which can not be filled with adhesive gold foil, or crystal gold. Even the crown of a molar tooth may be formed wholly of gold; when the crown is gone, leaving the root smooth and sound a little outside the margin of the gum.

Very considerable attention has been given to the forms of plugging instruments, and it is now demonstrated that there is far more in this particular than was conceived of by the operators of but a little while ago. The condensing instruments, containing from two to eight definite, perfectly formed, sharp points, prove to be far superior for condensing gold of any quality to any forms hitherto employed. Formerly, large, blunt points were used; now, perfectly sharp ones, and the results demonstrate their superiority. Owing to the conditions of gold to which we have referred, of which advantage is taken, and the improvement of plugging instruments, a fine finish is far more easily obtained than by the former proceeding. In the mechanical departments of the profession, the progress is evident more in the gradual improvement of old methods, than in new and startling discoveries. While processes that commend themselves to us for their intrinsic excellencies, though, perhaps, not yet fully developed, are growing in favor, the test of time and experience is as certainly consigning other methods to early and merited neglect. The continuous gum work and gutta percha are favorable as examples of this fact.

The improvements in continuous gum work consist chiefly in the more perfect condition of the materials and improved modes of manipulation. The formulas, as originally used, were subject to many essential imperfections, as want of strength, checking, blistering, etc. The materials now used are more refractory, and require a heat approaching nearly to that used in the manufacture of porcelain teeth. If we are correctly informed, borax is entirely dispensed with, which obviates the liability to blistering upon repeated heatings, besides imparting greater strength to the body. Formerly, much reliance was placed upon the strength imparted to the work by substantial linings, but a greater degree of strength is now claimed for the body alone, and backings are only used as a means of support for the teeth while the material is being applied. Heretofore the tedious and difficult operation of soldering rims to the plate was resorted to; now, they are either struck up, or the following plan adopted, which proves more expeditious and convenient:

Plain plates are swaged, the teeth arranged, slight linings adjusted, and the body or base applied, leaving a margin of uncovered plate around the borders of the width desired for the rim. The base is then backed and the uncovered marginal portions of the plate carefully turned over with a pair of pliers, and pressed down upon the edge of the porcelain base.

The material for the gum enameling is then applied flush with the overturned edge or rim of the plate and fused. If carefully done, the work when finished will present a neat and finished appearance, in all respects equal to any other method of riming, and is a great saving of time and labor. Much of the work at present done consists in enameling the entire lingual face of the plate. In other cases, a duplicate plate is soldered to the primary one, and made continuous on the inside of the teeth with the gum body. Or the two plates are made of equal dimensions, leaving a space between them equal in width to the air chamber, filling in the space not soldered with body through an opening in the gum plate, corresponding in size to an ordinary chamber, thus converting Cleaveland's chamber into Gilbert's. These processes all bear the marks of improvement, and render this style of work more desirable.

That style of constructing artificial dentures entirely from teeth upon porcelain, introduced by Dr. Loomis, we believe has not been used to any considerable extent beyond the limits of northern Ohio. It is generally, so far as the committee is aware, esteemed impracticable, from the difficulty and uncertainty of estimating with sufficient accuracy the amount of shrinkage in the process of baking. Further improvement will doubtless be made, which will increase its claims upon the attention of the profession.

The same may with propriety be said of Dr. Satterthwaite's style, which is somewhat similar to the one just mentioned, and liable to the same objections.

In regard to the use of gutta percha as a base for artificial dentures, especially for permanent work, the common sentiment of the profession, based upon sufficient tests of its utility, will relieve the committee from the duty of passing judgment. It has been "weighed in the balance and found wanting." That the material may meet the expectation of its friends in some cases of temporary work is possible, and that it may be moulded into many appliances subservient to the wants of the profession in many particulars, is quite certain. Beyond such purposes, we have no present grounds for belief in its utility.

Last, though certainly not least, if faith is to be placed in the testimonials of credible witnesses, comes "Cheoplasty.' Whether the claims set up for this style of work by the patentee and those who have adopted it in their practice, are to be fully realized, remains, probably, a question to be determined by time and a more extended experience in its use. These constitute the touchstone by which the qualities of all important inventions in Dentistry have heretofore been tested, and it is not likely that Cheoplasty will escape this ordeal. Some methods have gone down beyond the hope of renovation, while others are acquiring fresh claims to confidence in their practicability and usefulness.

The committee is in possession of no facts in relation to this style of work, not open to the professional public, and are, therefore, not any more capable of passing upon its merits. We shall give only a brief synopsis of what is claimed for it, viz: that it may be sufficiently attenuated to fit it for practical use without being clumsy or unmanageable in the mouth, and yet possess all the strength and stiffness of an ordinary gold plate. That it is indestructible in the mouth, undergoing no change of color, and unaccompanied by any appreciable metalic taste. That it is superior to metal, and equal to any other style of work in point of cleanliness, the several parts so incorporated as to exclude the lodgment of foreign matters subject to decomposition. Its susceptibility of a perfect and faultless adaptation to the mouth, in all cases where a true impression of the parts has been obtained, the material for the base being cast directly upon the plaster model. Its exemption from contraction in cooling. Its capability of being moulded and formed into any desirable shape. The ease and facility with which the piece may be constructed. Great economy of time in its construction. The facility with which it may be repaired, when broken or impaired by accident. The reduced cost of the materials employed, compared with other processes.

As the reasons for or against this style of work will probably be fully elicited during the discussion of one of the questions before the association, we refrain from further comments.

The committee are unadvised of any marked or specific improvements in the manufacture of porcelain teeth. Indeed, there seems to be but little room for progress left in this particular. Manufacturers are, doubtless, steadily advancing in this department from year to year, in the structure, coloring and translucency of their teeth, as well as modifications in form, to suit the varying exigencies of practice.

Besides the items of special improvements already mentioned, there are, doubtless, others not enumerated. To do so and describe them, would extend this report beyend reasonable limits. The committee would, therefore, close it, indulging the hope that the profession, taking encouragement from the past, will continue to extend their researches into the yet unexplored fields of science and art, so that from year to year it shall increase in usefulness, honor and influence, and stand as an honored branch of the healing art, more and more perfectly vindicated in the good opinion and respect of the world.

J. Richardson,
J. Taft,
H. R. Smith.

